# Monolayer Colloidal Crystals by Modified Air-Water Interface Self-Assembly Approach

**DOI:** 10.3390/nano7100291

**Published:** 2017-09-25

**Authors:** Xin Ye, Jin Huang, Yong Zeng, Lai-Xi Sun, Feng Geng, Hong-Jie Liu, Feng-Rui Wang, Xiao-Dong Jiang, Wei-Dong Wu, Wan-Guo Zheng

**Affiliations:** 1 Research Center of Laser Fusion, China Academy of Engineering Physics, Mianyang 621900, China; yexin@caep.cn (X.Y.); zengy91@163.com (Y.Z.); sunlaixi@yahoo.com (L.-X.S.); gengfeng@mail.ustc.edu.cn (F.G.); hongjie3713@163.com (H.-J.L.); wfr2000@163.com (F.-R.W.); 2 Hefei Institute of Physical Science, Chinese Academy of Science, Hefei 230031, China; 3 Hefei Institute of Physical Science, University of Science and Technology of China, Hefei 230026, China; 4 Science and Technology on Plasma Physics Laboratory, Research Center of Laser Fusion, China Academy of Engineering Physics, Mianyang 621900, China; wuweidongding@163.com; 5 IFSA Collaborative Innovation center, Shanghai jiao tong University, Shanghai 200240, China

**Keywords:** colloidal microspheres, colloidal crystal, monolayer, self-assembly

## Abstract

Hexagonally ordered arrays of polystyrene (PS) microspheres were prepared by a modified air-water self-assembly method. A detailed analysis of the air-water interface self-assembly process was conducted. Several parameters affect the quality of the monolayer colloidal crystals, i.e., the colloidal microsphere concentration on the latex, the surfactant concentration, the polystyrene microsphere diameter, the microsphere polydispersity, and the degree of sphericity of polystyrene microspheres. An abrupt change in surface tension was used to improve the quality of the monolayer colloidal crystal. Three typical microstructures, i.e., a cone, a pillar, and a binary structure were prepared by reactive-ion etching using a high-quality colloidal crystal mask. This study provides insight into the production of microsphere templates with flexible structures for large-area patterned materials.

## 1. Introduction

Two-dimensional (2D) micro/nanostructured arrays have received considerable attention recently because of their important applications in areas such as biosensors [1,2,3], magnetic materials [4,5,6], diffraction gratings [7,8], photonic crystals [9,10], and subwavelength structures [11,12,13,14,15,16,17]. Ordered monolayer colloidal microspheres form on certain substrates with a hexagonal close-packed (HCP) alignment [18]. These ordered microsphere arrays are termed 2D colloidal crystals. 2D colloidal crystals are often used as masks to fabricate 2D ordered micro/nanostructured arrays [19,20,21]. Colloidal crystal technology has been developed recently because of the successful synthesis of monodispersed colloidal spheres. High-order 2D colloidal crystals have been fabricated on various substrates, i.e., silicon, glass, and SiC, by different self-assembly methods [1,22,23,24], such as drop-coating [25], spin-coating [1], dip-coating [22], hierarchical self-assembly [26], and electrophoretic deposition [27]. This improves the potential applications of these materials or nanostructures.

Drop-coating self-assembly has been developed because of its low cost, high efficiency, and simplicity [25]. Close-packed three-dimensional colloidal crystals can be fabricated easily using this method. However, it is difficult for controlling the number of layers by drop-coating. In realistic applications of monolayer colloidal crystals, a major obstacle is how to fabricate large-area colloidal monolayers without defects.

Many authors have fabricated 2D colloidal crystals by continuous convective assembly, which is also termed dip-coating technology [28]. This method was developed originally by Nagayama et al. [22]. During continuous convective assembling, monodisperse polystyrene (PS) microspheres were dispersed in deionized water initially. To prevent aggregation, sodium dodecyl sulfate (SDS) was added to the suspension of microspheres before self-assembly. Monolayer colloidal crystals formed on the hydrophilic substrate after vertical immersion and lifting up from the microsphere suspension. The number of layers was controlled mainly by adjusting the concentration of microspheres and the lifting speed from the suspension. However, the dip-coating method is unsuitable for fabricating large-area samples because of the formation of stripes on the surface.

The spin-coating method has often been used to fabricate monolayer colloidal crystals. First, a drop of suspension is applied to a substrate. Then, the substrate is spun at a suitable speed, in which a suspension of fine microspheres spreads rapidly over a rotating substrate [29]. However, this method is more useful for several-layered colloidal crystals. It is difficult to approach large-scale, low-defect-density colloidal crystals by using this method. Voids and multilayered areas form continuously on the monolayer colloidal crystal samples. These features are a major limitation for this technique.

To fabricate high-quality monolayer colloidal crystals, the Langmuir-Blodgett (LB) method was used to transfer monolayer colloidal crystals from the air-water interface to the substrate. This makes large-scale colloidal-crystal-domain formation easy [30]. However, special expensive equipment, i.e., an LB machine, is needed in this method. The effect of pH was investigated in previous work to improve the quality of monolayer colloidal crystals [31]. Mineral acid was added to adjust the pH. However, residual impurities of mineral acid on the substrate are unavoidable. To avoid PS microsphere dispersion and to improve the quality of monolayer colloidal crystals, SDS has been added to microsphere suspensions. This helped the second self-assembly process moderately [32].

We present a modified air-water self-assembly approach to produce high-quality larger-scale colloidal monolayers. This method does not require special expensive equipment or additional compounds. First, PS microspheres were self-assembled on a silicon wafer by spin-coating. Then, the coated wafer was immersed in water for a second self-assembly on the air-water interface. Finally, floating monolayer colloidal crystals were transferred to a target substrate. A low concentration of PS microspheres was used to avoid PS microsphere agglomeration on the wafer. Once they were immersed into water, loose monolayer colloidal crystals floated on the water surface. SDS was added to change the water surface tension to facilitate a second self-assembly of loose monolayer colloidal crystals on the water surface. The second step was improved by a self-designed mechanical device, which makes the immersion of initial substrate-coated PS microspheres steady. This helps form high-quality monolayer colloidal crystals. A series of parameters that affects the self-assembly process was investigated. They include the concentration of PS microspheres, the SDS concentration, the PS microsphere diameter, and the PS microsphere polydispersity. Thus, large-area, high-quality, and transferable monolayer colloidal crystals were achieved in this work.

## 2. Experimental Details

### 2.1. Materials

All chemicals, including potassium persulfate (99%, Aladdin, Aladdin Industrial Corporation, Shanghai, China), styrene (99%, Aladdin), ethanol (99.95%, Aladdin), sulfuric acid (98%, Aladdin), hydrogen peroxide (35%, Aladdin), and SDS (99%, Aladdin) were used as purchased. Silicon wafer (20 mm × 20 mm) was used as a substrate for the fabrication of monolayer colloidal crystals. The silicon substrates were treated in piranha solution (3:1 *v*/*v*, 98% H_2_SO_4_/35% H_2_O_2_) at 90 °C for 30 min before use.

### 2.2. Synthesis of Monodisperse PS

Monodisperse PS microspheres were from Duke Scientific Corporation (Shanghai, China). Their size distribution was less than 3%. To investigate the effect of size distribution for flaws in colloidal crystals, we also polymerized monodisperse PS microspheres [33]. PS microspheres were polymerized in a five-neck flask. The temperature was 80 °C. Reactants included styrene and potassium peroxodisulfate. The mixed solution was stirred at 300 rpm for 24 h. The PS microspheres were dispersed in deionized water. The size distribution of the microspheres that were fabricated in this experiment is shown in Figure S1.

### 2.3. Fabrication of 2D Array

The diameters of the PS microspheres that were used varied from 80 nm to 750 nm. PS microspheres were dispersed in water solutions at different concentrations. The silicon wafer that was used as a substrate was cleaned by immersion in piranha solution (7:3 concentrated H_2_SO_4_:30% H_2_O_2_) for 30 min at 90 °C, and rinsed repeatedly with deionized water (18.2 MΩ cm^−1^). The substrates were dried in nitrogen before use [34].

The prepared solutions (80 μL) of different concentrations were deposited on a hydrophilic wafer and distributed using a spinner (SC-1B Photoresist spinner, Beijing Jinshengweina Technology Co. Ltd., Beijing, China). The silicon wafer was spun at 600 rpm for 10 s and at 3200 rpm for 1 min. After drying the dispersion, the wafer was immersed slowly in Milli-Q water at 15 °C. The immersion speed was controlled by a stepper motor at ~1 cm/min. After the wafer had been immersed in the deionized water, unordered monolayer colloidal crystals formed on the water surface. To consolidate the microspheres, 20 μL SDS solution of different concentrations was added into the water to adjust the surface tension. Monolayer colloidal crystals that floated on the surface were transferred to the receiving substrate by dip-coating. Finally, high-order monolayer colloidal crystals were obtained on the substrate when all the water had evaporated.

To demonstrate the versatility of this colloidal crystal, we fabricated 2D arrays on the silica substrate. Etching was carried out with reactive-ion etching (RIE, Oxford Plasmalab 80Plus-RIE, Oxford, UK). A pillar array was etched by a fluorine-RIE process. Gas from the fluorine-RIE process was a mixture of Ar:CHF_3_ (150 SCCM:15 SCCM). The chamber pressure was 1.2 Pa and the etching time was 20 min. The Radio Frequency (RF) power was set to 100 W. Wet etching of tetrahydrofuran was introduced to remove the remaining PS microspheres. The cone and binary structure was etched using a 30:3:1 mixture of Ar (150 SCCM), CHF_3_ (15 SCCM), and oxygen (5 SCCM). The chamber pressure and RF power were set at 1.2 Pa and 100 W, respectively. All samples were cleaned by using ethanol before characterization.

### 2.4. Characterization

The distribution of microsphere diameters was measured by using a Mastersizer 2000 laser (Malvern Instruments Ltd, Malvern, UK) particle sizer. The morphology of the monolayer colloidal crystals templateand the structure as etched with RIE were examined by using a JSM-7401 scanning electron microscope (SEM, JEOL Ltd, Beijing, China). A thin layer of Au (~10 nm) was coated on the sample before imaging. The images of the monolayer colloidal crystals were analyzed by using fast Fourier transformation (FFT), and the duty factor of the microsphere was used to determine the degree of ordering.

## 3. Results and Discussion

**Figure 1:**
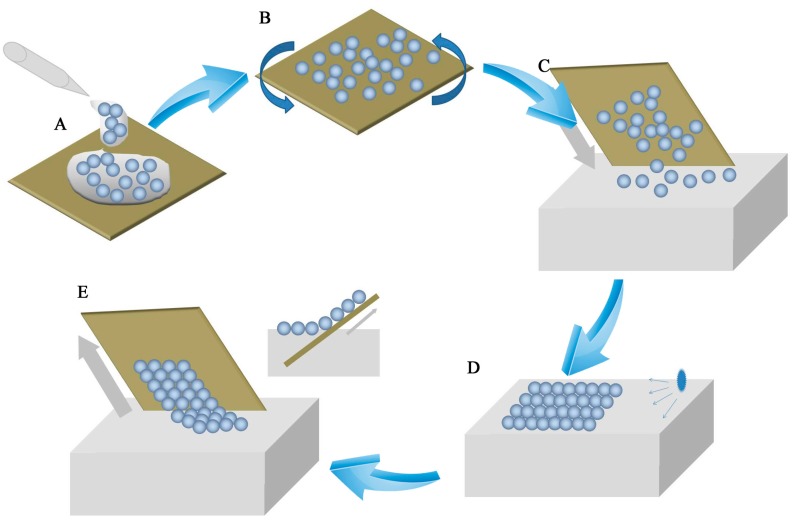
Schematic diagram of the air-water interface self-assembly method. (**A**) Drops of solution were applied on the wafer surface; (**B**) the wafer was spun to achieve a colloidal microsphere film; (**C**) the wafer with polystyrene (PS) microspheres was dipped slowly into deionized (DI) water. PS microspheres floated on the water surface; (**D**) PS microspheres self-assembled on the air-water interface when the surface tension was changed by using SDS; (**E**) a high-order colloidal crystal was transferred to the substrate.

As shown in Figure 1, the air-water interface self-assembly method included five steps. A drop of PS microsphere suspension was applied to the silicon wafer (Figure 1A). The silicon wafer was spun by a spin-coater to approach prior self-assembly (Figure 1B). PS microspheres that were spread on the wafer as unstable sub-monolayer colloidal crystals were separated. The PS microspheres were converted from the hydrophilic to the hydrophobic form. A wafer with PS microspheres was dipped slowly into deionized water (Figure 1C). Unstable sub-monolayer colloidal crystals floated on the water surface. Floating PS microspheres packed into dense colloidal crystals on the water surface. These sub-monolayer colloidal crystals consisted of separate PS microspheres. To obtain highly ordered monolayer colloidal crystals, the water surface tension was modified by using a SDS solution (Figure 1D). The change in tension lead to a highly order monolayer colloidal crystal self-assembly. In the last step, the floating monolayer colloid transferred from the water surface to the substrate by dip-coating (Figure 1E).

High-order monolayer colloidal crystal formation can be influenced by many experimental parameters, such as the PS microsphere concentration, the microsphere diameter, the interfacial tension of air-water, and the microsphere monodispersity. Apparently, a key point is to optimize each parameter to achieve highly ordered monolayer colloidal crystals. The first part that was investigated in this work focused on how the concentration of microspheres influences the monolayer quality.

**Figure 2:**
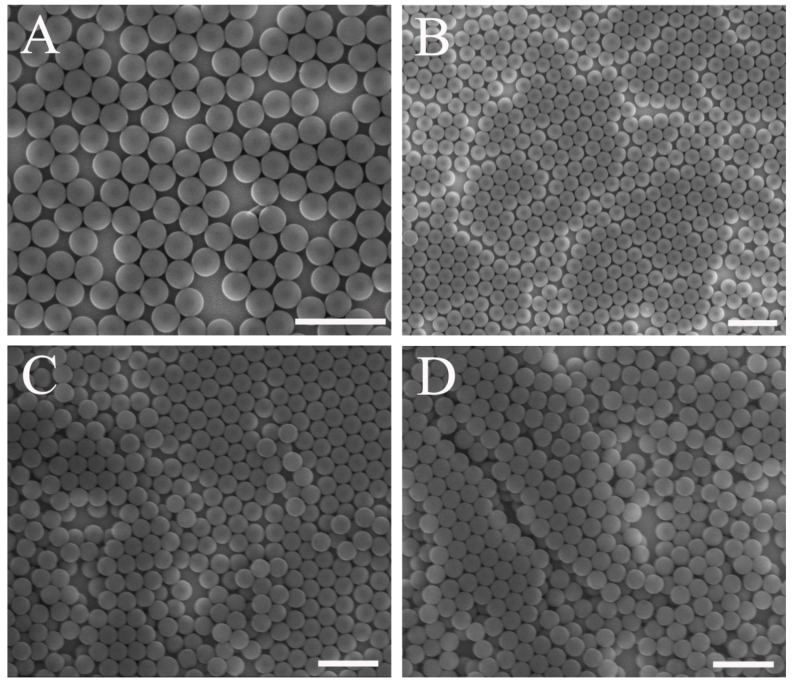
Colloidal crystals fabricated using PS microspheres with different concentrations. (**A**) 1 wt. %; (**B**) 2 wt. %; (**C**) 5 wt. %; (**D**) 10 wt. %. (Scale bar 1 μm. All colloidal crystals were already transferred to the substrate after the last step).

The effect of PS microsphere concentration on the monolayer colloidal crystals was investigated (Figure 2). The diameter of the PS microspheres was 250 nm. A different quality of colloidal crystals was obtained at different concentrations. Sparse microspheres formed on the substrate for a PS microsphere concentration of 1 wt. %, as shown in Figure 2A. This occurs because insufficient microspheres exist in the solution film. Figure 2B shows that the colloidal crystal was fabricated using PS microspheres with a 2 wt. % concentration. A sub-monolayer that consists of domains of HCP and disordered colloidal microspheres was visible on the substrate. Multilayer colloidal crystals were obtained on the substrate for a high concentration of microspheres. As shown in Figure 2C,D, domains of two and three layers of colloidal crystals were visible on the substrate, respectively. The SEM images in Figure 2C,D shows that multilayer flaws occurred at the second spin-coating step for high concentrations, as shown in Figure 1. As the microsphere concentration increased, a cluster formation emerged. The polarity of the PS microspheres changed during the drying of water after spin-coating. Colloidal microsphere films that float on the air-water interface exhibited a higher mechanical stability. As a result, no re-self-assembly occurred at the air-water interface during substrate immersion. Therefore, the ability to rearrange and re-self-assemble on the air-water interface is important for the formation of high-quality monolayer colloidal crystals. A similar result has been reported in another approach [35].

Sparse microspheres on the substrate allowed for microsphere floatation and maximal microsphere mobility at the air-water interface for optimal ordering when the substrate was immersed in water. A low concentration of microspheres would be more useful for high-order monolayer colloidal crystals. The unstable, sparsely distributed microsphere film that floated on the air-water interface shrunk when the surface tension changed on one side. High-order monolayer colloidal crystals were obtained as described in the next section.

**Figure 3:**
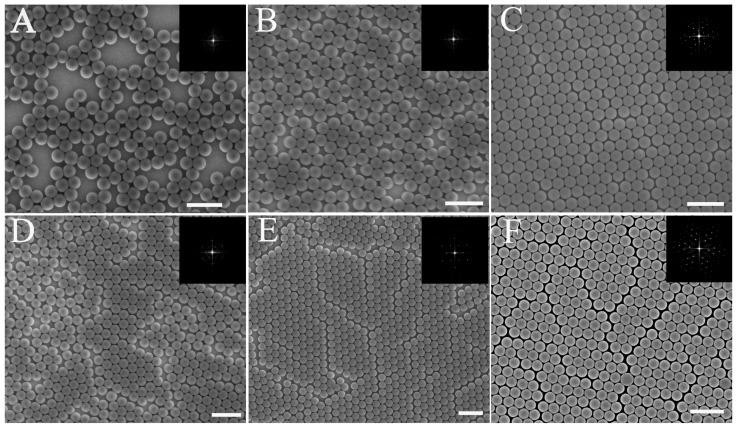
Influence of SDS concentration on monolayer formation for low PS concentrations (upper row, 1 wt. %) and high PS concentration (lower row, 2 wt. %). The SDS concentrations were (**A**,**D**) non-SDS, (**B**,**E**) 2 wt. %, (**C**,**F**) 4 wt. %. (Scale bar 1 μm, all colloidal crystals had been transferred to the substrate after the last step). The insets are the relevant fast Fourier transformation (FFT) images.

The key point of the air-water interface self-assembly method is the second self-assembly at the air-water interface. After silicon wafers with PS microspheres were dipped into deionized (DI) water, the PS microspheres floated on the water surface as disordered sub-monolayer colloidal crystals. A change in surface tension on the side of the floating sub-monolayer colloidal crystals pushed the separating PS microsphere to achieve a second self-assembly on the water surface. SDS was added into the microsphere suspension to change the surface tension. To elucidate the underlying mechanism, a detailed SDS effect on the quality of colloidal crystals was analyzed. Figure 3 shows a comparison of colloidal films with different concentrations of SDS solution. As discussed earlier, the low concentration of microspheres is positive for the high-order monolayer colloidal crystals. Therefore, a low concentration of microspheres were used in the subsequent experiments.

From an analysis of several samples, we can divide colloidal crystals that were fabricated by this method into three types: (1) sparsely distributed colloidal crystals, (2) a monolayer with few line defects, and (3) an ordered microsphere monolayer. As demonstrated previously, PS microspheres that floated on the water surface did not self-assemble if no change in surface tension occurred. Therefore, after the colloidal film was transferred to the substrate, a sparse microsphere distribution and domains of HCP colloidal microspheres on the parent substrate formed, as shown in Figure 3A,D. Here, colloidal crystals as shown in Figure 3A–E were fabricated using PS spheres with microsphere concentrations of 1 wt. % and 2 wt. %, respectively.

The FFT and duty factor were used to estimate the degree of uniformity. The detail of the method is shown in the supplementary materials. The colloidal film with PS microspheres that floated on the surface of the water was unstable. To optimize the morphology of the monolayer colloidal crystals, surfactant was applied to change the surface tension. The abrupt change in surface tension resulted in a shrinking of the colloidal film and a consolidation of the colloidal crystals. This treatment significantly improves the quality of the monolayer colloidal crystals during the second self-assembly process at the air-water interface. As shown in Figure 3B,C, these monolayer colloidal crystals were prepared using different concentrations of surfactant. SDS (20 μL of 2 wt. % and 4 wt. %) was added into the water. The respective duty factors of the microsphere as calculated from Figure 3A–C were 55.9%, 80.16%, and 90.3%. This result shows that sparse microspheres were compressed because of surface tension modifications. From the SEM image and the FFT results (inset), a high-order monolayer colloidal crystal can be obtained with an increase in SDS concentration, as seen in Figure 3C. The sparse colloidal microspheres or small clusters floated on the water surface with a high mobility. A high concentration of SDS results in a large change in surface tension. The large change in surface tension pushes the colloidal microspheres into a high-order monolayer colloidal crystal. Figure 3B shows that the degree of uniformity is low, although the microsphere duty factor is close to the ideal factor (90.6%). Therefore, the 4 wt. %-concentration of SDS is most suitable for solutions with a 1 wt. %-concentration of microspheres. It is not the long-range hexagonal ordering from the SEM image and the FFT shown in Figure 3B. Therefore, a degree of uniformity can be assessed by combining the FFT and the duty factor of the microsphere. Even if a high concentration of SDS was applied in the water, some line defects are visible on the monolayer colloidal crystal that was fabricated by using a 2 wt. % PS microsphere solution, as shown in Figure 3E,F. The FFT image inset in Figure 3D shows many dispersive points as a circle, which implies a low degree of uniformity. Bright Fourier frequency spectra are visible from the FFT image insets in Figure 3D,F. The hexagonal ordering of the sample is clearly visible from the FFT result. Line flaws are visible in the SEM image. However, some line flaws do not affect the permutation orientation of the hexagonal order. This explains why the Fourier frequency spectra of Figure 3E,F are bright, despite the presence of line flaws. In this case, the duty factor of the microsphere is necessary to assess the degree of uniformity. The microsphere duty factors as calculated from Figure 3D–F are 80.1%, 81.9%, and 85.1%, respectively. Therefore, the degree of uniformity of these samples is lower than that of the ideal monolayer colloidal crystals, and the SDS does not work as well for higher solution concentrations due to the microsphere immobility in higher concentration solutions. Therefore, a change in surface tension can improve the monolayer colloidal crystal quality, especially for low concentrations of PS microspheres.

**Figure 4:**
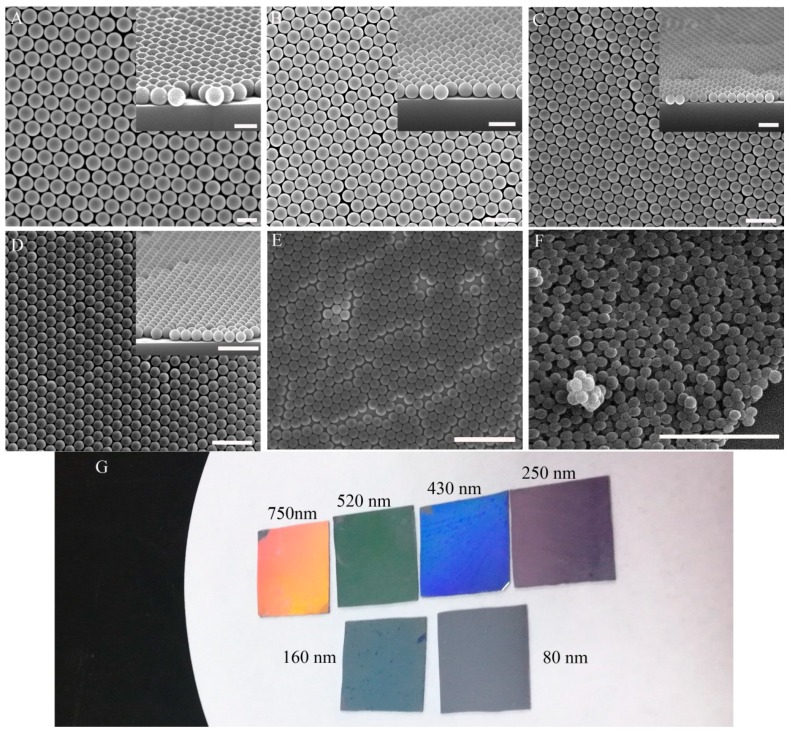
Monolayer colloidal crystals with different diameters. (**A**) 750 nm; (**B**) 520 nm; (**C**) 430 nm; (**D**) 250 nm; (**E**) 160 nm; (**F**) 80 nm. (Scale bar 1 μm. All colloidal crystals were already transferred to the substrate after the last step). (**G**) Large-scale monolayer colloidal crystals with different diameters. High-order monolayer colloidal crystals are visible in the photograph with a bright, uniform color.

The diameter of the PS microspheres also influences the quality of the monolayer colloidal crystals. The surface tension and capillary force figured prominently during self-assembly. The surface tension can be controlled by SDS. The capillary force is related to the microsphere diameter. A smaller microsphere diameter resulted in a stronger capillary force. To best understand the relationship between microsphere diameter and monolayer colloidal crystal quality, PS microspheres with different diameters were self-assembled to form monolayer colloidal crystals. The PS microsphere diameters were 80, 160, 250, 430, 520, and 750 nm. Figure 4A–E show the high-quality colloidal crystals that were prepared from 750-nm, 520-nm, 430-nm, 250-nm, and 160-nm particles, respectively. The inset image is the tilted view SEM micrograph of the monolayer colloidal crystal. Figure 4G shows corresponding digital-camera pictures of large-scale monolayer colloidal crystals with different diameters. The uniformly bright color from the centimeter scale means that high-quality colloidal crystals formed on the substrate. The colloidal crystals as shown in Figure 4F were prepared from PS microspheres with an 80-nm diameter. Nanoparticles aggregated easily during spin-coating and drying of water. Multilayers and clusters formed on the water surface during wafer immersion with nanoparticles. After these nanoparticles had been transferred to the substrate, a cluster formation emerged. Therefore, it is difficult to prepare high-quality monolayer colloidal crystals by using small microspheres (with a diameter below 100 nm).

**Figure 5:**
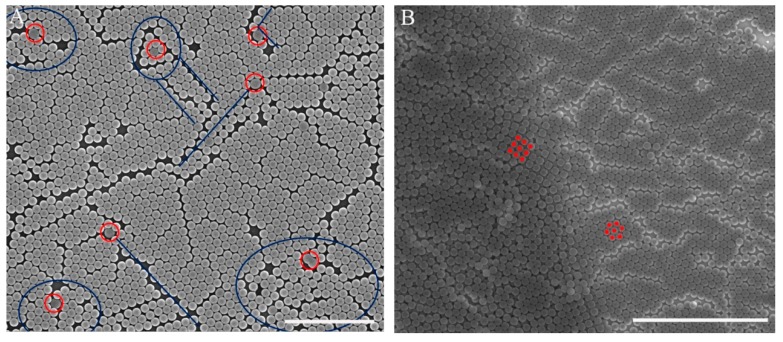
(**A**) Influence of defect precursors on monolayer colloidal crystal; (**B**) alignment boundary of monolayer and two layers. (Scale bar 3 μm).

To the best of our knowledge, monodispersed PS microspheres are very important for high-order colloidal crystals, especially for monolayer colloidal crystals. We investigated how defect precursors (e.g., non-spherical particles and microspheres with different diameters) affect the quality of monolayer colloidal crystals. As shown in Figure 5A, monolayer colloidal crystals were fabricated by 250-nm diameter polydispersed PS microspheres. The defect precursors were marked by a red circle. The defects included line defects and a disordered area, and they were marked by blue lines and circles, respectively. The image shows that the line defect often resulted from PS microspheres with different diameters. Disordered areas were often observed around non-spherical particles. The HCP structure had a stable distribution because of its low energy. A face-centered cubic structure was often observed at the boundary of the monolayer and the two layers, as shown in Figure 5B.

**Figure 6:**
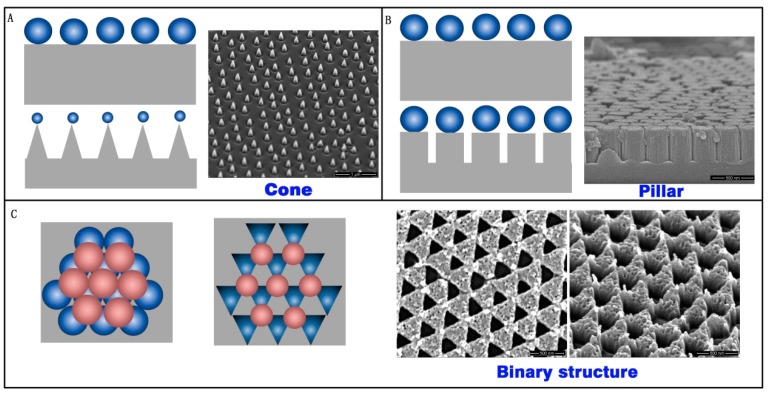
(**A**) Cone structure; (**B**) pillar structure; and (**C**) binary structure fabricated with reactive-ion etching (RIE) using colloidal crystals.

As a mask of the array structure, colloidal crystals are high quality, inexpensive, and have a low time-cost. Three types of array structures were prepared with RIE by using colloidal crystals as a mask. A cone array, pillar array, and binary array were fabricated on the silica substrate, as show in Figure 6. The cone array was prepared with CHF_3_ and O_2_ RIE fluorine-oxygen plasma etch silica, using PS microspheres. The PS microsphere diameter decreased during etching, and the cone profile finally appeared (Figure 6A). The pillar structure formed on the substrate after the CHF_3_ RIE process. The microsphere diameter did not decrease during pure fluorine-plasma etching. The pillar profile was achieved in this case, as shown in Figure 6B. If two layers of colloidal crystals exist on the substrate, the structure will form a binary array after CHF_3_ and O_2_ RIE, as shown in Figure 6C.

## 4. Conclusions

We demonstrated experimentally that a modification of surface tension, PS microsphere concentration, microsphere polydispersity, and PS microsphere diameter are important points for close-packed monolayers of PS microspheres. SEM imaging and FFT analysis were applied to estimate the degree of uniformity of monolayer colloidal crystals that were fabricated under different conditions. According to research, large-scale, high-quality monolayer colloidal crystals can be prepared by using PS microspheres with different diameters from 160 nm to 750 nm. It is difficult to obtain monolayer colloidal crystals from small microspheres (microsphere diameter < 100 nm) because of the increase in cluster formation with a decrease in microsphere diameter. The possibility of obtaining large-scale, high-quality monolayer colloidal crystals by changing the surface tension is simple for implementation. Sparsely distributed microspheres on the initial substrate as prepared by using a lower concentration of microspheres were better at a high concentration. The monolayer colloidal crystals can be transferred to a non-planar substrate. Therefore, this method could be applied to fabricate various masks for applications. Three types of structures were prepared by RIE and by using our high-quality colloidal crystal mask in the final part.

## Supplementary Materials

The following are available online at http://www.mdpi.com/2079-4991/7/10/291/s1, Figure S1: Size distribution of microsphere fabricated in our experiment, Figure S2: Schematic of duty factor of microsphere. D is diameter of microsphere.
