# Supplementary material for: Monolayer Colloidal Crystals by Modified Air-Water Interface Self-Assembly Approach
Source: Nanomaterials (Basel). 2017 Sep 25;7(10):291. doi: 10.3390/nano7100291 (PMC5666456; doi:10.3390/nano7100291)
Supplement: Supplementary file 1 [file nanomaterials-07-00291-s001.pdf]

Article

Monolayer Colloidal Crystals by Modified Air-Water Interface Self-Assembly Approach

Xin Ye[1], Jin Huang[1,4,5※], Yong Zeng[1,] , Lai-Xi Sun[1], Feng Geng[1], Hong-Jie Liu[1], Feng-Rui Wang[1], Xiao-Dong Jiang[2*], Wei-Dong Wu[2], Wanguo Zheng[1, 3]**

[1]Research Center of Laser Fusion, China Academy of Engineering Physics, Mianyang, Sichuan 621900, P.R.China

[2]Science and Technology on Plasma Physics Laboratory, Research Center of Laser Fusion, China Academy of Engineering Physics, Mianyang, 621900, P.R. China

[3]IFSA Collaborative Innovation center, Shanghai jiao tong University, Shanghai, 200240, P.R. China

[4] Hefei Institute of Physical Science, Chinese Academy of Science, Hefei 230031, China;

[5]University of Science and Technology of China, Hefei 230026, China

※Corresponding author: (Jin Huang)

huangjin3011@163.com

*Corresponding author: (Xiaodong Jiang)

jiangxdong@163.com

**Corresponding author: (Wanguo Zheng)

yehanwin@mail.ustc.edu.cn

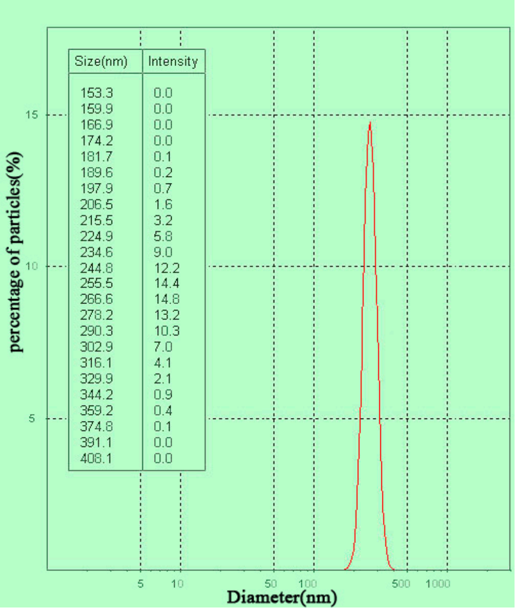

Figure S1. Size distribution of microspheres fabricated in our experiment.

Fast Fourier transform (FFT) of an SEM image often has been used to study the structure of arrays. The long range order can be demonstrated by FFT of an SEM image. However, it is difficult to investigate the degree of uniformity of colloidal crystals by FFT. The duty factor of microspheres in a unit area can be used to access the degree of uniformity of monolayer colloidal crystals. As shown in Figure S2, for ideal situations, the duty factor of a microsphere is $f =$

$\frac{2\pi(D/2)^2}{\sqrt{3}D\times D}=\frac{\pi}{2\sqrt{3}}$ for hexagonal order in a unit area with $\sqrt{3}D\times D$. We believe that the duty factor of the hexagonal order is largest for monolayer colloidal crystals. Therefore, the duty factor of microspheres is a parameter which can be used to investigate the degree of uniformity of monolayer colloidal crystals. Here in this experiment, statistics concerning the number of microspheres was determined by image process software (Image-Pro plus 6). The experimental duty factor of microspheres is f_e = number of microspheres × area of great circle of microsphere / total area. As discussed above, the ideal factor is 90.7%. The closer the experimental factor was to the ideal factor (90.7%), the higher the degree of uniformity of the monolayer colloidal crystals.

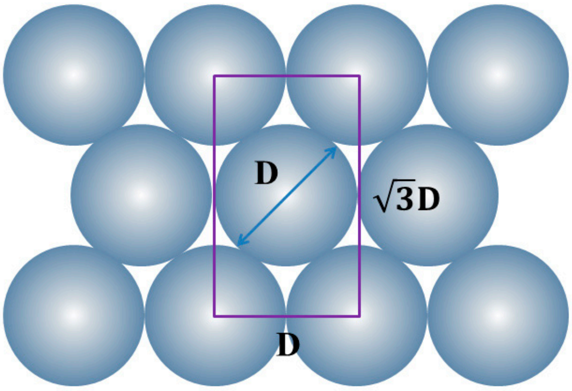

Figure S2. Schematic of the duty factor of microspheres. D is the diameter of the microspheres.
